# Management of patients with neuromuscular disorders at the time of the SARS-CoV-2 pandemic

**DOI:** 10.1007/s00415-020-10149-2

**Published:** 2020-08-17

**Authors:** Gianluca Costamagna, Elena Abati, Nereo Bresolin, Giacomo Pietro Comi, Stefania Corti

**Affiliations:** 1 Neuroscience Section, Department of Pathophysiology and Transplantation (DEPT), Dino Ferrari Centre, University of Milan, Via Francesco Sforza 35, 20122 Milan, Italy; 2 Neurology Unit, Foundation IRCCS Ca’ Granda Ospedale Maggiore Policlinico, Via Francesco Sforza 35, 20122 Milan, Italy; 3 Neuromuscular and Rare Diseases Unit, Foundation IRCCS Ca’ Granda Ospedale Maggiore Policlinico, Via Francesco Sforza 35, 20122 Milan, Italy

**Keywords:** Neuromuscular disorders, COVID-19, Telemedicine, Vaccine, Pandemic, Disease-modifying therapies, Neuromuscular disorder centres, Ventilatory support

## Abstract

The novel Coronavirus disease-19 (COVID-19) pandemic has posed several challenges for neuromuscular disorder (NMD) patients. The risk of a severe course of SARS-CoV-2 infection is increased in all but the mildest forms of NMDs. High-risk conditions include reduced airway clearance due to oropharyngeal weakness and risk of worsening with fever, fasting or infection Isolation requirements may have an impact on treatment regimens administered in hospital settings, such as nusinersen, glucosidase alfa, intravenous immunoglobulin, and rituximab infusions. In addition, specific drugs for SARS-CoV2 infection under investigation impair neuromuscular function significantly; chloroquine and azithromycin are not recommended in myasthenia gravis without available ventilatory support and prolonged prone positioning may influence options for treatment. Other therapeutics may affect specific NMDs (metabolic, mitochondrial, myotonic diseases) and experimental approaches for Coronavirus disease 2019 may be offered “compassionately” only after consulting the patient’s NMD specialist. In parallel, the reorganization of hospital and outpatient services may change the management of non-infected NMD patients and their caregivers, favouring at-distance approaches. However, the literature on the validation of telehealth in this subgroup of patients is scant. Thus, as the first wave of the pandemic is progressing, clinicians and researchers should address these crucial open issues to ensure adequate caring for NMD patients. This manuscript summarizes available evidence so far and provides guidance for both general neurologists and NMD specialists dealing with NMD patients in the time of COVID-19.

## Introduction

**Fig. 1:**
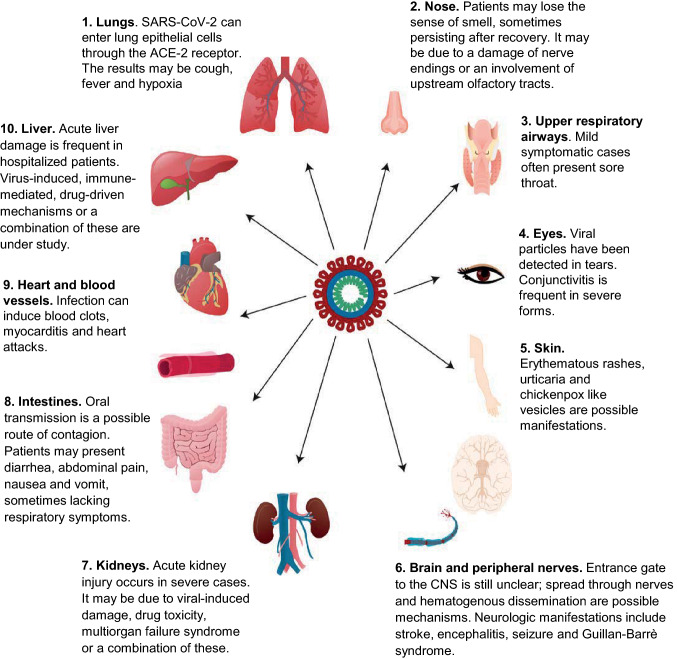
Systemic involvement in SARS-CoV-2 infection. The figure shows the different organs and systems possibly involved in coronavirus disease-19 (COVID-19) infection and it summarizes some pathophysiological and clinical-related features. The virus can spread beyond the lungs and the respiratory tract, affecting the gastrointestinal, cardiovascular, renal, and nervous system. Eyes and skin may be involved as well. Possible routes of contagion include aerial, oral, and conjunctival transmission

Since the end of December 2019, the severe acute respiratory syndrome virus 2 (SARS-CoV-2) pandemic has claimed the lives of more than 400,000 individuals worldwide (https://coronavirus.jhu.edu/map.html). Symptomatic SARS-CoV-2 infection causes a wide spectrum of symptoms (referred to as “Coronavirus Disease 2019”, COVID-19), such as fever, dry cough, and fatigue in milder cases and systemic manifestations in severe disease courses (Fig. 1). In parallel, SARS-CoV-2 infection poses a greater risk for old, oncologic, and immunosuppressed patients, which also include many individuals with hereditary and acquired neuromuscular disorders (NMD) that may already present increased risks due to the underlying disease (see “Risk assessment and stratification” section). As already reported in other papers, the first phase of the SARS-CoV-2 pandemic has seen the overwhelming access of COVID-19 patients to the Emergency Departments prompting an urgent reorganization of personnel and facilities in worst-hit areas, such as Wuhan [1], New York [2, 3] and Lombardy [4, 5]; this reallocation of resources has also imposed changes in the short- and mid-term management of NMD outpatients and non-urgent cases, favouring the use of at-distance approaches [6]. As the first wave of the pandemic is progressing in many countries, we need adaptive strategies for the post-pandemic phase regarding NMD patients. In this sense, sharing the experience gained from centres directly involved in COVID-19 management may help the neurologic community facing common challenges.

Specifically, first-hand experiences regarding neuromuscular centres have been reported [6] and different general recommendations for NMD patients are available (e.g. World Muscle Society), but the literature on the management of non-infected and infected NMD patients during the pandemic is limited. In addition, some issues regarding NMD patients remain open: (1) drugs used compassionately for SARS-CoV-2 patients (e.g. azithromycin, chloroquine, and hydroxychloroquine) are contraindicated in some NMD patients (myasthenic patients) and can cause NMD-related complications (rhabdomyolysis, toxic neuropathies, and myopathies); (2) the use of anaesthetics, intubation, and mechanical ventilation is the standard of care for severe SARS-CoV-2 cases in intensive care units (ICU), but it poses additional risks for NMD patients with bulbar involvement; (3) neuromuscular disorders centres should reorganize to offer the highest level of care while securing best standards of safety for the NMD patients.

Here, we aim to review the management of NMD patients with and without SARS-CoV-2 infection, focusing on COVID-19 therapies, COVID-19 drug-related complications, and ventilatory support, sharing the experience from our NMD centre in Lombardy, proposing our view for the role of NMD centres and NMD specialists during the post-acute pandemic phase and addressing the future challenges that neurologists dedicated to neuromuscular disorders will probably encounter.

## The NMD patient without SARS-CoV-2 infection

### Risk assessment and stratification

Risk assessment and stratification are crucial to identify NMD patients who are at increased risk to suffer from severe COVID-19 complications. National and international neurologic and neuromuscular networks have produced guidance to help both NMD patients and families and their physicians to prevent and manage SARS-CoV-2 infection in this category [6–9]. Overall, these documents recognize that the risk of severe complications from COVID-19 is high or moderately high in most NMD patients, apart from those with the mildest forms.

Features associated with high or very high risk include weakness of respiratory muscles with a reduction of respiratory volumes, use of invasive or non-invasive ventilation devices, oropharyngeal weakness determining inefficient airway clearance, the use of immunosuppressive treatments and the presence of severe comorbid conditions and/or multiorgan impairment [8] (Table 1).

**Table 1:** Factors conferring a high or very high risk of developing severe COVID-19 complications

Muscular weakness of the chest and/or diaphragm, resulting in respiratory volumes less than 60% predicted
Use of non-invasive or invasive ventilation devices
Presence of tracheostomy
Presence of dysphagia and oropharyngeal weakness (reduced airway clearance)
Primary cardiac involvement
Risk of deterioration with fever, fasting or infection
Risk of rhabdomyolysis with fever, fasting or infection
Concomitant diabetes, obesity, neoplastic diseases, severe cerebrovascular diseases or severe heart diseases (heart failure, ischemic heart disease)

Some conditions, such as mitochondrial diseases, metabolic myopathies and myasthenia gravis, might also determine a worse outcome because of the risk of deterioration or rhabdomyolysis [10] secondary to fever and infections. In addition, patients receiving steroid treatment might undergo an adrenal crisis during fever or infection if steroid dosage is not adjusted [11]. Patients with conditions that do not affect respiratory and swallowing functions and whose immunocompetence is not affected by medications are generally not considered to be high risk [8]. However, there are additional risk factors that might add to the pre-existent neurologic condition and increase the risk in the single patient (Table 2).

**Table 2:** Additional risk factors increasing the risk of developing severe COVID-19 disease

Kyphoscoliosis
Highly-active immune-mediated neuromuscular disease
Mild respiratory muscle weakness
Other medical comorbidities: • Pulmonary diseases • Liver diseases • Neutropenia/lymphopenia • Renal diseases/impairment
Older age
Pregnancy (possible)
Concomitant additional neurologic diseases
Dependence from caregivers in hygiene, mobilization and feeding

Therefore, risk assessment should be carried out on a case-by-case basis. It is worth pointing out that highly-dependent patients that need help from caregivers in basic activities of daily living (BADL) present an increased risk due to their impossibility to isolate [12].

Following available guidelines, and in the opinion of the authors, NMD patients who do not present a high or very high risk of severe complications should follow general rules outlined in the World Muscle Society (WMS) guidelines [8]. Conversely, high-risk patients should be advised to practice “shielding”, that means not leaving their home and avoid any face-to-face contact and social situation unless extremely necessary [9].

### The role of neuromuscular centres

The SARS-CoV-2 pandemic has forced a quick reorganization of hospital facilities and outpatient services to address new patients’ needs. Local differences in response have depended mainly upon geographical localization of hospitals, patient population, distribution of sick patients, and the overall organization of the health care system [6, 13]. In our case, we may distinguish between two different phases of the response to the pandemic.

During the first days after the virus spread in our area, the need for intensive and sub intensive care beds in hospitals selected as “COVID Hubs” (appointed by the authorities), the shortage of PPE and the increased infection risk for patients imposed consistent changes in the management of our NMD patients. Elective or “non-urgent” patient visits were postponed to avoid unnecessary exposure for patients and clinical staff, favouring virtual encounters. Other services, such as electrodiagnostic tests, muscle, and nerve biopsies, were suspended unless urgent cases required further evaluations (such as new diagnosis of amyotrophic lateral sclerosis, myasthenia, myositis, or immune-mediated neuropathies). Ancillary services needed reorganization of personnel and resources: pulmonary assessments were stopped due to increased risk of viral shedding from tested patients; tests for dysphagia were performed for cases at increased risks of complications or unplanned hospitalizations; speech therapy and neuropsychological evaluations, whenever possible, were conducted on virtual platforms.

**Fig. 2:**
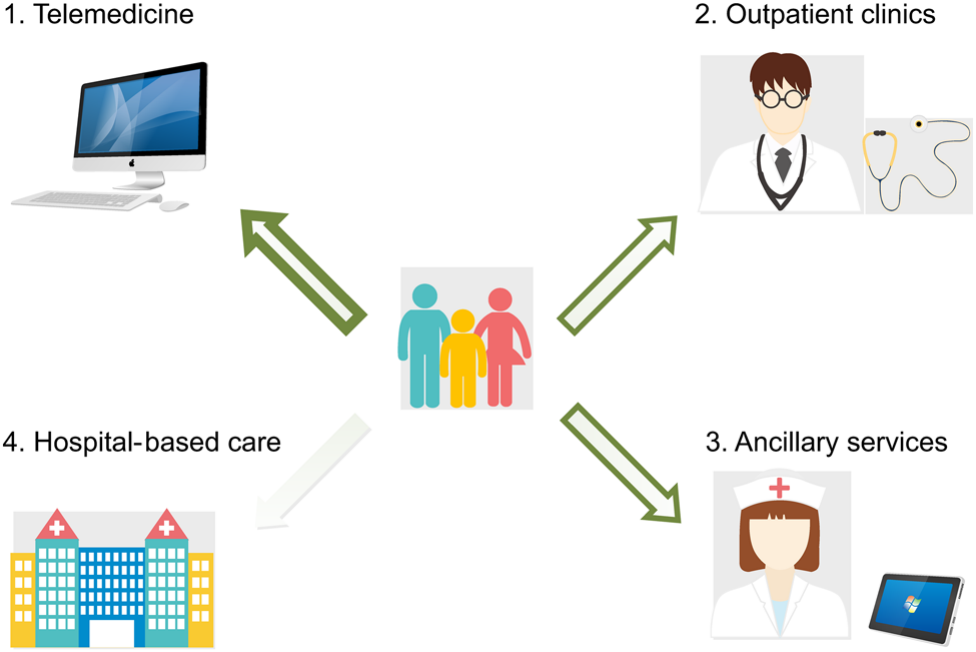
The four pillars of neuromuscular disorder centres and their function during the SARS-CoV-2 pandemic. This figure displays the four main organizational milestones that could improve the care of neuromuscular disorder (NMD) patients during the pandemic. The prominent use of telemedicine approaches (wide green arrow), if possible, can help to avoid unnecessary hospital visits for NMD patients. Ancillary services performed as much as possible with virtual platforms, such as pulmonary assessments, fluoroscopic swallowing studies, and neuropsychological evaluations, and outpatient clinics represent valuable alternatives to hospital visits (medium-width green arrows). NMD patients’ visits in hospital settings, particularly if dedicated to COVID patients, should be proposed more sporadically, (narrow-width green arrow), be preferred for low-risk NMD patients, and be provided following strict safety measures (see “The role of neuromuscular centre” section for more details)

In the second phase of the pandemic, when the curve of COVID-19 cases started to flatten and COVID-dedicated facilities were partially dismissed, we adopted new strategies for our NMD patients (Fig. 2). At the time of writing, the number of new daily cases in many western countries is decreasing (see COVID map link), but strategies to effectively resume subspecialty outpatients’ clinics and to reorganize hospital settings in the post-acute pandemic phase still lack. National and international recommendations, though heterogeneous, identify some higher-risk subjects that may need prolonged strict preventive measures even after the worst phases of the pandemic, such as adults older than 65, immunocompromised, and cancer patients (e.g. https://www.esmo.org/for-patients/patient-guides/cancer-care-during-the-covid-19-pandemic) (Tables 1 and 2). NMD patients at higher risks for SARS-CoV-2 complications should be included in this list (see “Risk assessment and stratification” section) . Depending on the number of new cases, COVID-dedicated hospitals should be reorganized to permit the management of outpatients and the provision of ancillary services, if possible; if not, referral centres for NMD patients should implement robust telemedicine services to allow for proper online visits and patient’s caring (see “Telehealth and telerehabilitation” section). We believe that low-risk individuals—who include most of NMD patients—could be assessed in COVID hospitals, using proper precautions regarding both facilities and PPE; patients should be cared for in separate, non-COVID buildings, they should wait in uncrowded waiting rooms, and should hygienize their hands and wear face masks and gloves at the facility entrance, similar to measures adopted in previous epidemics [14].

An initiative that may help neuromuscular centres to reorganize patient care is the use of quality-of-life surveys sent to NMD patients to follow up on their feelings and needs and consequently modulate appropriate responses, as already assessed in immunocompromised patients [15]. In parallel, nationwide surveys may help to quickly identify NMD patients who suffered from severe SARS-CoV-2 infection in other regions/states of the country and promptly manage possible complications—such as ventilation-associated pulmonary damage, critical-illness myopathies, and neuropathies and emerging COVID-related neurologic manifestations [16–18]—that were reported during the SARS-CoV-1 pandemic in 2002–2004 [19].

### Telehealth and telerehabilitation

The pandemic has prompted a reorganization of rehabilitation services as well. Rehabilitation patients’ admission has been reduced in different countries to sustain increased hospital bed capacity and home-based rehabilitation services have been suspended or offered at-distance [13]. Alternatives for face-to-face visits include telemedicine that refers to the provision of health care services using technology, such as text, e-mail, telephone, and video [20, 21]. Despite different studies assessing the use of teleneurology—including telerehabilitation—for patients with chronic neurologic conditions exist [22, 23], the available literature on NMD patients is scarce.

Overall, three unblinded studies on the use of teleneurology in ALS patients reported satisfaction among patients, caregivers, and health-care providers [24–26]. Two studies point out the same level of care [27] and comparable survival [28] in ALS patients treated with at-distance approaches versus in-person visits. In addition, virtual approaches seem to reduce emergency room access and acute hospitalizations in ALS [28]. Telehealth services for the initial and follow-up evaluation in patients with myopathies are associated with comparable results between virtual and in-person neurology consultations [29]. A teleneurology study based on a small cohort (*n* = 4) of patients with facioscapulohumeral muscular dystrophy suggested that this approach is considered acceptable by patients and it correlates with a reduction of hospital admission [30]. In another study, a telehealth-based clinical grading scale for patients with polyneuropathies shows high sensitivity (98%), specificity (91%), and acceptable concordance with in-person examination [31]. Other studies on the suitability of this remote evaluation system for polyneuropathies are emerging [32]. Considering telerehabilitation for neurologic patients, a Cochrane review highlights low-level evidence for telerehabilitation to improve disability, fatigue, and quality of life in MS patients [33]. A similar comprehensive analysis lacks for NMD patients. However, a small retrospective study evaluating telehealth-guided rehabilitation on 26 patients with mixed neuromuscular disorders reported improvement in cognitions, self-care, quality-of-life, and mobility [34].

During the pandemic, the need for prevention and mitigation strategies has moved telemedicine towards the front lines of clinical practice. Professional societies are sharing guidelines on the correct implementation of telehealth approaches for neurological patients (e.g. https://www.aan.com/siteassets/home-page/tools-and-resources/practicing-neurologist--administrators/telemedicine-andremote-care/20-telemedicine-and-covid19-v103.pdf) [35] and reports of their rapid deployment are emerging [20, 36]. The use of remote neurologic examination requires adaptation and inventiveness. Muscle strength might be evaluated by movement against gravity, assessed with weights or resistive tools, or by functional activities, such as standing up from a sitting position with or without assistance. The nuances of deep tendon reflexes may be challenging via virtual approaches; however, a caregiver, if present, may be instructed to grossly evaluate briskly increased, reduced or asymmetric reflexes by tapping with fingers or objects [20]. A recent example of a neurologic examination at-distance comprises a suspected diagnosis of SARS-CoV-2 infection in a young patient with Charcot–Marie–Tooth polyneuropathy undergoing post-surgical telerehabilitation sessions [36]. Disease and non-disease-specific scales have been proposed for patients with NMD. Particularly, Revised ALS Functional Rating Scale (ALSFRS-R) for amyotrophic lateral sclerosis and Myasthenia Gravis Activities of Daily Life (MG-ADL) for myasthenia gravis are validated, disease-specific scales potentially useful for remote evaluations. Other proposed, non-disease-specific scales include Myo-FRS for myopathies and Nerve-FRS (N-FRS) for neuropathies, which may be practical tools for assigning a functional score [37].Altogether, these results suggest that teleneurology and telerehabilitation for NMD patients, though at their infancy, are feasible and valuable approaches during the pandemic.

### Disease-modifying therapies

Different expert panels [38] and international societies (e.g. Muscle Dystrophy Society) have already provided general recommendations regarding disease-modifying therapies used in NMD patients that may increase the risk of infection or a more severe disease course. As a general rule, NMD patients should continue to take their medications, unless differently instructed by an NMD specialist via telephone or online. These medications include also newly FDA and/or EMA approved drugs, such as enzymatic replacement therapy (ERT) for Pompe disease, antisense oligonucleotides (ASO) for Duchenne Muscular Dystrophy (DMD) patients with deletions amenable to exon 51 skipping, splicing-modulator ASO and gene therapy for Spinal Muscular Atrophy (SMA), and messenger-RNA interfering molecules for transthyretin (TTR) amyloidotic neuropathy. The challenge with these drugs lies more within the highly specialised setting required for their administration, involving recurrent visits to hospital tertiary centres, rather than within specific contraindications of the administered compounds.

Several NMDs recognise immune-mediated pathogenesis. The extent to which immunosuppressive drugs can contribute to severe forms of SARS-CoV-2 infection is still unclear. Depending on the regional pandemic burden, patient compliance, and caregivers’ support, NMD specialists may want to consider a reduction of the dose of some immunosuppressive drugs or shift to less immunosuppressive alternatives for high-risk patients [6]. This decision should imply a careful discussion with the patients on the overall risk, specific benefits, and potential adverse events of the new treatment. Regarding corticosteroid therapy, abrupt withdrawal may induce flair of the underlying condition, requiring higher doses of steroids and increasing the risk for hospitalization [39].

Currently, no evidence suggests that immunoglobulin therapy (intravenous or subcutaneous), plasma exchange, or Fc receptor antagonists (efgatirgimod, currently tested in clinical trials for myasthenia gravis (MG)) [40] may increase the risk of infection or a severe disease course.

Immunomodulant therapies currently used in NMD patients may help against SARS-CoV-2 infection. Some severe SARS-CoV-2 cases with acute respiratory insufficiency may be associated with complex immune dysregulation and excessive activation of inflammatory pathways [41]. At the time of writing, some cytokine inhibition strategies are currently being tested for the treatment of COVID-19, such as Tocilizumab and Eculizumab, but their efficacy in COVID-19 patients is still unclear; several clinical trials (NCT04288713 for eculizumab; NCT04317092; NCT04310228 and others for tocilizumab) are currently ongoing worldwide.

An adequate provision of drugs represents another key issue for NMD patients. Frequent visits to pharmacies may expose them to unnecessary risk. However, during the pandemic, many pharmacies have expanded their services, waiving fees for home-delivery, and extending refill periods [42].

## The NMD patient with SARS-CoV-2 infection

### SARS-CoV-2 infection in NMD patients

Clinical syndromes associated with SARS-CoV-2 infection have been classified by the World Health Organization (WHO) as uncomplicated disease, mild pneumonia, severe pneumonia, acute respiratory distress syndrome (ARDS), sepsis, and septic shock [7]. In case of symptoms suggestive for SARS-CoV-2 infection, NMD patients should follow local health authorities' policies, call their health care provider to receive guidance on treatments, testing, and monitoring [9], and contact their neuromuscular specialist to discuss possible drug dosages adjustments and potential treatments. The decision to temporarily withhold or reduce immunosuppressant medication should be discussed with the neuromuscular specialist, and should never be done by the patient without consultation [8, 38]. Most patients affected with COVID-19 have a mild disease course and should continue the current best practice standard of care [38]. Patients receiving corticosteroids might require an increase in treatment doses due to the risk of infection-triggered hyposurrenalism [38]. However, decisions regarding treatment de-escalation should be individualized according to the severity of infection, the risk for disease exacerbation, and the intensity of immunosuppression [38].

Inpatient admission should be avoided when possible, but it should not be delayed if necessary [8]. Indeed, patients with baseline reduction of breathing capacity and pulmonary volumes are especially at risk of rapid worsening of the disease. We suggest preparing an Emergency card stating specific respiratory and treatment needs of the single patient, including settings for ventilatory devices [9]. Furthermore, we suggest that myasthenic patients have ready with them a list of medications to avoid and that all patients with muscular involvement ensure they have a card stating pharmacological requirements for general anaesthesia [9].

Patients, caregivers, and their physicians and neurologists should bear in mind that, in an emergency setting, such as during the SARS-CoV-2 pandemic, hospitals may have to apply a ceiling of care based on age and pre-existing conditions [43–45]. In a setting of scarce resources (e.g. insufficient ICU capacity, unavailability of CPAP devices, etc.), triaging on ICU admission may have been instituted [43–45]. However, the differences between outcomes and treatability of different neuromuscular conditions might not be known to all emergency and intensive care specialists, thus neurological consult should be sought and promptly provided whenever doubts arise regarding the proper treatment of NMD patients. Even in the case of critically ill NMD patients, the role of the NMD specialist remains crucial. To guarantee a fair provision of intensive care to NMD patients, the NMD specialist should establish a close collaboration with respiratory and intensive care specialists. As there is great variability among NMD patients in terms of prognosis, treatability, and disease stage, these patients should not be denied life-saving treatments only based on their disability and without consultation with a neurologist or NMD specialist [45]. To facilitate decisions in case neurological consult is not promptly available, the French Rare Health Care for Neuromuscular Disorders Network developed a list of neuromuscular diseases usually carrying a good prognosis, that could be eligible for admission in ICU, and, for other conditions not on the list, criteria suggesting a favorable outcome in case of ICU admission [45].

Conditions with a good prognosis for recovery include autoimmune and congenital myasthenia, metabolic myopathies, inflammatory myopathies without severe systemic damage (in particular, pulmonary fibrosis), muscle channelopathies, and most neuropathies, hereditary or acquired [45]. The positive criteria for a good prognosis for intensive care in these patients are no major cardiac or respiratory damage and no major disability.

For other patients, the criteria in favor of resuscitation/ICU admission are [45]:

- Neuromuscular pathologies with slight progression
- Respiratory functions being minimally impaired and stabilized
- Mild and stabilized heart disease
- Absence of severe thoracic deformities/severe contractures preventing ventral decubitus
- Absence of multisystemic impairment and comorbidities
- Preservation of autonomy for everyday life acts and/or social environment to supplement daily life tasks (e.g., presence of a family and caregivers)

Similarly, patients with end-stage or incurable diseases should be encouraged to discuss with their loved ones their wishes regarding resuscitation and intubation, and, if necessary, to write them down beforehand.

Limited literature exists on the clinical course and recovery of NMD patients with SARS-CoV-2 infection. A small case series of 5 immunosuppressed MG patients showed high variability of disease severity and outcome [46]. Three of them developed severe respiratory insufficiency following the infection and required intubation or high-flow oxygen, whereas two had a milder disease course. Four of them had a favourable outcome. Indications of therapy discontinuation in immunosuppressed MG patients during the infection are uncertain [38]. Although this patient population lacks a long-term follow-up, in which an exacerbation may present, mycophenolate was discontinued in two patients without experiencing a subsequent flair of the underlying disease after recovery [46]. In some MG patients, SARS-CoV-2 pulmonary infection may directly induce an MG crisis [46, 47].

### Oxygen therapy and ventilatory support

WHO recommends that all patients with SARS-CoV-2 severe acute respiratory infection (SARI) are started immediately with supplemental oxygen therapy at 5 L/min and that flow rates are titrated to reach target SpO_2_ $\ge$ 90% [7]. However, NMD patients should be closely monitored and early invasive or non-invasive ventilatory support should be considered in NMD patients who develop interstitial pneumonia, as hypoxemia might quickly lead to pump failure due to exhaustion and weakness of respiratory muscles, even in previously compensated patients, and hypercapnia may worsen disease course.

Hypercapnia is not a classic feature of SARS-CoV-2 pneumonia, and its appearance might signal the onset of respiratory muscle weakness [7]. Furthermore, extensive interstitial pneumonia or acute respiratory distress syndrome (ARDS) usually does not respond to oxygen therapy alone, even when oxygen is delivered via a face mask with reservoir bag, as a hypoxemic respiratory failure in ARDS is usually the result of intrapulmonary ventilation-perfusion mismatch or shunt and therefore requires mechanical ventilation [7]. Indeed, acute hypoxemic respiratory failure requiring respiratory support was the main reason for ICU admittance in large cohorts of COVID-19 patients [48–52]. The median arterial blood oxygen (PaO_2_) to fraction of inspired oxygen (FIO_2_) ratio on ICU admission spanned from 136 to 169 [48–50], very low levels, thus requiring high levels of Positive End-Expiratory Pressure (PEEP). Thereby, the need of endotracheal intubation (ETI) and invasive mechanical ventilation in published studies on critically ill COVID-19 patients ranged from 30% (Wuhan, China) [52], 42% (Wuhan, China) [51], 47% (Wuhan, China) [50] to 71% in Washington State, US [49] and 88% in Lombardy region, Italy [48].

Conversely, ICU patients that could be managed with non-invasive ventilation were, respectively, 62% [52], 56% [51], 42% [50], 19% [49] and 11% [48]. As regards NIV, data on Middle East Respiratory Syndrome (MERS) patients suggest a high failure rate [53]. Moreover, NIV carries the risk of widespread diffusion of exhaled air and airborne viral transmission, although recent reports show that newer systems with good interface fitting might reduce this risk [54, 55]. It is recommended that patients receiving a trial of NIV remain in a monitored setting, with the possibility of rapid ETI in case of acute deterioration or lack of improvement [7].

ETI and mechanical ventilation remain the mainstay of treatment for unstable patients with ARDS and acute respiratory insufficiency. High levels of PEEP are generally required in COVID-19 patients with ARDS [48], and in case of severe ARDS, prone positioning is recommended [7]. As regards NMD patients, anaesthetic risk varies widely, as it depends mainly on baseline muscular and ventilatory function and the presence of comorbidities [56]. In some cases, the atrophy of masticatory muscles and limited mobility of the cervical spine may complicate ETI procedure [57]. In these cases, intubation should be performed following guidelines for difficult airway management [58]. Another reason for concern in this kind of patients is the potential of side effects from neuromuscular blockers and anaesthetic agents. Depolarizing muscle relaxants (succinylcholine) are contraindicated in NMDs because of the risk of fatal hyperkalemia and rhabdomyolysis [59]. Non-depolarizing muscle relaxants (e.g. rocuronium, rapacuronium, atracurium) should be used with caution and require dose reduction and careful titration in some categories of NMDs (myotonic disorders, myasthenia gravis, Lambert–Eaton myasthenic syndrome, spinal muscular atrophy, polymyositis, and immune-mediated neuropathies) that present impaired production of choline acetyltransferase and acetylcholinesterase, and reduced concentration of acetylcholine at the endplate [59]. As concerns sedation, intravenous anaesthetics are preferable to volatile agents in most neuromuscular patients [59].

### Treatments for SARS-CoV-2 and NMD diseases

Several drugs are currently undergoing clinical trials for use against COVID-19 and, in many countries, compassionate use for patients with severe disease has been approved (Table 3).

**Table 3:** Experimental treatments for SARS-CoV-2 infection

Drug	Results so far	Potential NMD-relevant side effects
Chloroquine (CQ)/Hydroxychloroquine (HCQ)	Open label studies of HCQ + azithromycin found an increased rate of viral load reduction or disappearance and clinical amelioration in most patients [60, 61] Randomized study of high vs. low dose of CQ failed to detect benefits (small sample size?) but higher CQ dosage not recommended because of potential cardiac toxicity [62]	QTc interval prolongation, favoring fatal arrhythmias, such as ventricular tachycardia and torsade de pointes, especially when combined with other QTc-prolonging drugs [63] CK elevation common Long-term use associated with risk of developing toxic neuromyopathy [64] and with onset or worsening of MG [65]
Lopinavir/Ritonavir (LPV/RTV)	Randomized trial found it did not improve outcome compared to SSC alone	QTc interval prolongation, favoring fatal arrhythmias, such as ventricular tachycardia and torsade de pointes, especially when combined with other QTc-prolonging drugs Risk of toxic myopathy in association with statins
Remdesivir	61 patients with severe COVID-19 were treated with remdesivir on a compassionate use basis: 68% of patients showed improvement, 15% worsened [66]	Myalgias in healthy controls [67] Elevation in liver enzymes [66]
Azithromycin	Open label studies of HCQ + azithromycin found increased rate of viral load reduction or disappearance and clinical amelioration in most patients [60, 61]	QTc interval prolongation Risk of worsening MG
Tocilizumab (TCZ)	Decrease in CRP levels in five patients receiving two or more TCZ administrations	Elevation in liver enzymes
Eculizumab (ECZ)	Clinical amelioration and drop in inflammatory markers in 4 critically ill patients treated with ECZ [68]	Myalgias and arthralgias Elevation in liver enzymes

*CK *creatine kinase,* CQ *chloroquine,* CRP *C-reactive protein, *ECZ *eculizumab,* HCQ *hydroxychloroquine,* LPV/RTV *lopinavir/ritonavir,* MG *myasthenia gravis, *SSC* standard supportive care,* TCZ *tociluzumab

Chloroquine (CQ) and its less toxic derivative, hydroxychloroquine (HCQ), have been developed as antimalarial drugs; nowadays, they are used also for chronic rheumatic diseases, such as systemic lupus erythematosus (SLE) and rheumatoid arthritis (AR). These drugs also show antiviral activity [69], and recent reports have drawn attention to a possible beneficial effect against SARS-CoV-2 at high doses [70–74]. One open-label study and one randomized (high vs. low dose) study were conducted, but definitive evidence regarding its efficacy is still lacking [61, 62, 75]. Lopinavir/Ritonavir (LPV/RTV) is an antiviral drug used for the treatment of HIV. A randomized Chinese trial found no efficacy of LPV/RTV compared to standard supportive care in patients with severe COVID-19 pneumonia [76], but other studies are currently ongoing (www.clinicaltrials.gov). Remdesivir is a novel nucleotide analogue developed for the treatment of Ebola and Marburg viruses. Remdesivir was shown to have in vitro antiviral activity against SARS-CoV-2 in vitro [73] and, in a cohort of patients with severe COVID-19 treated with remdesivir, the majority showed clinical improvement [66]. However, a randomized study conducted in China failed to show statistically significant differences between treated and placebo group, although a slightly faster clinical improvement was observed in the former group [77]. Further studies are now underway to better clarify Remdesivir efficacy (www.clinicaltrials.gov). Azithromycin is a macrolide antibiotic that may be able to suppress inflammation [78] and be effective as adjunctive therapy in ARDS [79]. It is now being studied as an adjunctive treatment for patients with COVID-19, with apparently positive results, but validation in randomized trials is needed [60, 61]. Tocilizumab is a monoclonal antibody targeting interleukin-6 receptor (IL-6R), inhibiting signal transduction and, thus, counteracting the effects of pro-inflammatory interleukin-6 (IL-6), that was shown to be involved in inflammatory storms in severe COVID-19 [80]. For this reason, the use of Tocilizumab was advocated in severe COVID-19 patients, and many clinical trials are now ongoing (www.clinicaltrials.gov), although published reports so far are scarce and anecdotal [81, 82]. Eculizumab is another monoclonal antibody directed against complement component 5 (C5), a member of the complement cascade, inhibiting its cleavage into C5a and C5b, both with proinflammatory and prothrombotic properties. The use of Eculizumab for treatment of severe COVID-19 is currently being investigated in a clinical trial (NCT04288713), and preliminary results on 4 critically ill patients were favourable [68]. Notably, Eculizumab has recently been studied as a therapeutic agent in generalized MG [83, 84].

The most dangerous complication that may arise during short-term treatment with such agents is QTc prolongation and arrhythmias [62, 63]. In addition to CQ/HCQ, many drugs that are now used against COVID, such as azithromycin and lopinavir/ritonavir, hold potential for QTc prolongation [85], which may already be altered in NMD patients, such as individuals with myotonic dystrophy [86]. The Canadian Heart Rhythm Society has released guidelines to minimize the risk of drug-induced ventricular arrhythmias [85]. In this document, they recommend performing baseline electrocardiography (ECG) in patients with an increased risk of rhythm alterations. If QTc is moderately prolonged, optimization of medications and electrolytes may permit therapy, while in patients with markedly prolonged QTc these therapies should be avoided [85]. CQ and HCQ are also associated with CK increase and, in long-term treatments, with the development of toxic neuropathy and myopathy [64, 87]. Furthermore, reports describe drug-induced myopathies and rhabdomyolysis in patients treated concomitantly with LPV/RTV and statins [88, 89]. For this reason, we recommend a careful risk/benefit assessment before use in myopathic patients, and, in case of treatment with these agents, regular monitoring of serum CK levels. HCQ and azithromycin also have the potential to cause the onset or worsening of myasthenia gravis, and thus use in these patients should be carefully evaluated [65, 90]. We do not recommend using these drugs as prophylaxis in NMD patients, as their prophylactic efficacy has not been proven and it may lead to serious toxicity [38].

Treating the underlying NMD disease in SARS-CoV-2 patients may be challenging. One report describes the therapy of an MG exacerbation presenting with dysphagia, neck weakness, and diplopia during the infection. Anand et al. [46] administered intravenous immunoglobulin (IVIg) (2 g/kg/die) and increased dose of steroids (from 2.5 mg every other day at basal to 20 mg per day during the infection). In another report, a SARS-CoV-2 case with ETI experienced an MG crisis involving the proximal upper and lower extremities [47]. This patient was treated with prednisone (increased from 20 to 40 mg twice daily), pyridostigmine, and IVIg (0.4 g/kg for five days). Due to a worsening of the proximal weakness of upper and lower limbs, another course of IVIg (650 mg/kg for two days) was performed. In both cases, the outcome was favourable.

Overall, the literature on NMD patients with SARS-CoV-2 infection is limited in number and general considerations are difficult. Best management for MG patients with SARS-CoV-2 may represent a dilemma and the use of large registries may prove helpful to improve care for this patient population.

## Concluding remarks

In this work, we have reviewed the existing literature regarding the management of NMD patients with and without SARS-CoV-2 infection, addressing the concerns and challenges on the recommendations on disease-modifying therapies, the use of oxygen and ventilatory support, the contraindications and complications of drugs used in COVID-19 patients, and the implementation of telehealth approaches for NMD patients. Besides, we have shared our experience from Lombardy, one of the SARS-CoV-2 worst-hit areas worldwide, our thoughts on the role of NMD specialists in the pandemic and our view on the current and future role of NMD centres, hoping to help the Neurological community in this common effort against the coronavirus.

Different reports on SARS-CoV-2 impact on neurologic patients have been published in different countries with unprecedented speed and collaboration among centres, but several questions regarding NMD patients remain unanswered. Given the possibility of subsequent waves of the pandemic, we will need a robust reorganization of neuromuscular centres, where the role of telemedicine will be prominent. We will have to stay vigilant on COVID-19 infection- and COVID-19 therapy-related NMD complications, patients with pre-existing NM diseases and new drugs that may worsen specific NMD conditions.

Finally, a strong collaboration among centres and institutions in the Neurology community will be fundamental and will provide the data to help patients with NM disorders; sharing near real-time information, using virtual communication platforms, receiving constant follow-ups from patients in the form of online surveys and updating uniform national and international guidelines may prove valuable in the mid-term period.

Now more than ever, the old saying holds: “no man is an island”.

## Availability of data and materials

All data generated or analysed during this study are included in this published article (and its supplementary information files).
